# Preventing the ends from justifying the means: withholding results to address publication bias in peer-review

**DOI:** 10.1186/s40359-016-0167-7

**Published:** 2016-12-01

**Authors:** Katherine S. Button, Liz Bal, Anna Clark, Tim Shipley

**Affiliations:** 1 Department of Psychology, University of Bath, Bath, BA2 7AY UK; 2 BioMed Central, London, UK

**Keywords:** Publication bias, Peer review, Results-free review, Transparency

## Abstract

The evidence that many of the findings in the published literature may be unreliable is compelling. There is an excess of positive results, often from studies with small sample sizes, or other methodological limitations, and the conspicuous absence of null findings from studies of a similar quality. This distorts the evidence base, leading to false conclusions and undermining scientific progress. Central to this problem is a peer-review system where the decisions of authors, reviewers, and editors are more influenced by impressive results than they are by the validity of the study design. To address this, *BMC Psychology* is launching a pilot to trial a new ‘results-free’ peer-review process, whereby editors and reviewers are blinded to the study’s results, initially assessing manuscripts on the scientific merits of the rationale and methods alone. The aim is to improve the reliability and quality of published research, by focusing editorial decisions on the rigour of the methods, and preventing impressive ends justifying poor means.

## Introduction

Psychology has received much criticism of late, with classic findings failing to replicate, and high-profile cases of scientific fraud [24, 45]. Psychology is not alone. The evidence of unreliable findings across biomedical and social sciences is compelling [2, 15, 20, 36, 42]. There is a surfeit of studies reporting significant positive results (typically, *p* < 0.05), often from studies with small sample sizes, or other methodological limitations, and a conspicuous absence of the corresponding null findings from studies of a similar quality. This distorts the evidence base, increasing the proportion of false positive findings, and leading to biased estimates in meta-analyses.

Central to the problem is the peer-review system, and the role it plays in perpetuating biases in the published record; generally, authors, reviewers, and editors prefer results which show support for tested hypotheses and are prejudiced against submitting or publishing inconclusive or null findings. Rosenthal famously referred to this as the file drawer problem [33]; statistically significant findings which support the alternative hypothesis are published, while those studies with inconclusive or negative results languish in the author’s file drawer, hidden from peer and public awareness.

As will be discussed, there are many factors that bias the decision-making of authors, reviewers and editors throughout the publication process to the detriment of a reliable evidence base. In the absence of external pressures, the simple human desire for seeking information that supports one’s beliefs, and ignoring that which does not [1, 26], means authors are more likely to find, and reviewers to believe, evidence that confirms accepted theories. There are also differences in interpretability of positive and null findings (compounded by common design flaws such as having low statistical power) which mean that positive results can be misguidedly seen to overcome methodological weakness that would be critical for a null finding.

The bias for positive results is further exacerbated by the external influence of a competitive research culture. Publications are the prime currency for advancing academic careers [43], and where editorial decisions are seen to favour positive results, researchers are encouraged to adopt practices to boost their chances of finding positive results [38]. These practices often increase the risk of findings being false positive or inflated estimates, and thus further undermine scientific progress [5]. However, in the competition for publication, this risk is either ignored, or accepted as a price worth paying.

In order to improve the quality and reliability of published research, the criteria determining publication must be aligned with those for conducting rigorous scientific practice. The purpose of scientific enquiry is to estimate the presence and size of causal associations, and results from studies designed and conducted to the highest standards of scientific rigour will provide the most reliable and informative estimates. Thus, for the optimal advancement of science it seems logical that decisions regarding what to publish would be better based on judging quality, rather than results [11]. One way to achieve this would be ‘results-free’ review, where results are hidden from editors and reviewers, forcing reviewer reports and editorial decisions to be based on the scientific rigour of the study design alone.

This month *BMC Psychology* launches a pilot to trial a new ‘results-free’ peer-review process, to address the problem of bias in the editorial process. Editors and reviewers will be blinded to the study’s results, and decide whether to accept or reject manuscripts based on the scientific merits of their rationale and methods alone. There are multiple insidious ways in which the fixation on positive results biases decision to the general detriment of science, and as outlined below, ‘results-free’ review has the potential to address many of them.

### Publication bias

Publication bias is the term for what occurs whenever the research findings in the published literature differ systematically from the population of all studies completed in a given area [34]. Publication bias arises from the decisions of investigators, reviewers, and editors to submit or accept manuscripts for publication based on certain study characteristics. This would be beneficial if decisions were made solely on study quality [11]. However, publication decisions are most influenced by the direction or strength of the study finding; strong results clearly in favour of the study hypothesis are overrepresented, while studies reporting mixed or null findings are underrepresented.

Psychologists provided some of the first empirical evidence that the literature was biased towards positive results [10, 18, 26, 37, 39–41]. In 1959, Sterling found that of all the articles which used tests of significance published in 4 journals, 97% found in favour of the alternative hypothesis. However, despite psychologists’ early awareness of the dangers of such a publication bias, psychology has been relatively slow to intervene and, in a similar analysis in 2010, over 92% of psychology/psychiatry papers were still found to claim support for their tested hypothesis [12], suggesting that the degree of publication bias has remained high.

Basing decisions to publish on the nature of a study’s results is wasteful. It distorts the evidence available for policy makers and other key stakeholders, leading to false conclusions which can have severe consequences. In the biomedical literature, this can put patients at risk if the published evidence falsely suggests that ineffectual or harmful treatments work [17]. Selectively publishing positive results also hinders the incremental progression of science, and may explain the paucity of basic findings translating into clinical applications [21, 31, 32]. Many a PhD student has been demoralised to find they have wasted a year or more of their training trying to replicate and build on seemingly well-established findings only to find out that many others have also tried and failed, but their null findings were unpublished.

However, despite the undermining effects of publication bias on the evidence base, it persists for a variety of reasons. At a relatively simple level, there are asymmetries in the dominant model of statistical inference which mean that null findings are more difficult to interpret, and more afflicted by the limitations of poor study design, than positive results. Thus authors are less inclined to write them up and reviewers more inclined to reject. At a systemic level, career pressures to publish offer a sharp incentive to authors to favour writing up papers with the greatest chance of success, and under the current system of publication this will inevitably favour positive results.

### The problem of interpreting null results

A major contributing factor to both reviewer and author decisions to publish is the differences in interpretability of positive and null findings. Despite its many documented problems, Null hypothesis significance testing (NHST) remains the dominant framework for much experimental psychological research. However, there are asymmetries in the inferences one draws in this approach that mean null results are more difficult to interpret than positive ones. NHST is a hybrid of Fisher’s concept of null hypothesis testing [14], and the Neyman-Pearson concepts of Type I (α) and Type II error (β) and statistical power (1-β), but its application tends to lean most on Fisher’s concept of null hypothesis testing ([44]; [7], in press).

The first asymmetry arises in the strength of inferential claim. Obtain a positive result (*p* < 0.05) and one can boldly reject the null hypothesis and claim evidence of an effect. However, obtain a null result, and one has simply failed to reject the null hypothesis; one cannot claim evidence of no effect. The second and related asymmetry presents in the different weighting researchers give to the risk of type I and II errors. Text-book research designs adopt a 5% Type I error rate (*p* < 0.05), while accepting a higher Type II error rate of 20% (i.e., 80% power). In practice, however, the asymmetry is even greater - researchers ostensibly adhere to the 5% type I rate but seem to pay little mind to statistical power, and studies with power as low as 20% are common [5].

The impact this has on author and editorial decisions is best illustrated with an example: Suppose a researcher runs a series of studies with 20% statistical power (and thus a type II error rate of 80%), and sets the significance threshold at 5%. A null result is uninformative. The study design is so poor (in terms of having insufficient statistical power) that the researcher expects 80% of the studies to miss genuine effects. As a null result is more likely than not to be an (type II) error, the researcher decides it is not worth writing up. If, on the other hand, the researcher finds a result that passes the 5% significance threshold, they might convince themselves (and the reviewers) that despite the low power, the finding is worthy of publication as the chance of it being an (type I) error is only 5%. While in the case of a single study this decision making may seem reasonable, it is clearly problematic when considered across a population of studies.

The above example illustrates how the importance attributed to methodological limitations, such as low power, is highly influenced by the results. As methodological limitations tend to reduce a study’s sensitivity to detecting effects (via increasing standard errors), null results are often seen as an expected consequence of poor design. In contrast, finding a statistically significant result in a study of similar quality is often interpreted as a success, because the effect was found ‘*despite the limitations’* of small sample size, or measurement error. Indeed, passing the significance threshold may be seen as indicative of how large, or robust that effect must be [13]. Thus a third asymmetry arises in study quality; design limitations are seen to weaken the case for publishing a null result, while passing the 5% significance criterion can be seen as a golden ticket for dismissing away methodological concerns.

Perhaps because of the differences in interpretation, reviewers have been shown to be highly influenced by the direction and strength of effects [11]. On average, null papers take several months longer from the time of submission to eventual publication than positive papers (median, 1.1 vs 0.8 years; P = .04), suggesting that null results receive more criticism during the peer-review process [19]. This delay may stem from the increased difficulties of trying to persuade reviewers of the merits of null findings.

Reviewers have also been found to judge the methods and quality of null studies more critically than those of positive studies. Mahoney [26] randomly assigned referees to review 1 of 5 versions of a manuscript, all with identical introduction and methods sections, but different results and discussion sections (positive, negative, methods only, mixed results with positive discussion, mixed results with negative discussion) . The methods, data presentation, scientific contribution, and publication merit of manuscripts with positive results were rated as being nearly twice as high as manuscripts with negative results. Thus, negative findings seem to disproportionally and detrimentally affect appraisals of study quality and merit. This suggests that any attempts to base editorial decisions on methodological merit, are likely to be biased if the results are known.

Reviewers and editors act as the gatekeepers to publication, and may hinder the progress of null findings that contradict their beliefs. Researchers can become welded to certain theories or ideas, promoting the evidence that supports the scientific dogma, while dismissing that which does not. Examining sex bias in psychotherapy, Smith [40] found that while the published literature supported the widely held notion that the standards clinicians’ hold regarding mental health are biased against women, the unpublished data obtained from data requests was found to show the same degree of bias but in the opposite direction. Similar to those reporting null results, studies with results that contradict the scientific dogma may be less likely to be submitted or face more hurdles to persuading reviewers that they are worthy of publication.

### Authors’ decisions and career pressures

Analysing a discrete population of conducted studies (Time-Sharing Experiments in the Social Sciences, k = 221), Franco and colleagues found that strong results were 60% more likely to be written up, and 40% more likely to be published, than null results [16]. When asked why they choose not to write up their null findings, 15 out of the 26 authors who replied suggested it was in the belief that null results have little publication potential. Based on the asymmetries described above, the authors’ decisions not to pursue null papers seem reasonable given the uphill struggle null papers face during the review process.

Academics are under increasing career competition and peer-reviewed publications, citations, and grant funding are the prime currencies for advancing academic research careers. Over the past 30 years, the number of faculty positions in the US has remained relatively constant, but the number of PhDs awarded has increased substantially [35]. The competition for faculty positions is therefore fierce. Once secured, retaining a faculty position can be dependent on meeting key performance targets, and the main indicators of academic success are number of publications, journal impact factors and number of citations [43].

As has been discussed, in the current publication system, positive results are more easily published, especially those studies reporting large effects which, despite methodological limitations, are often published in high-impact journals. Indeed, meta-analyses have found that the degree of inflation in positive results correlates to the impact factor of the publishing journal, with highly biased results from small studies published in some of the highest impact journals [27]. In addition to being easier to publish in higher impact journals, positive results are also more likely to be cited once published, thus further increasing the incentives for authors to find them [25].

All of this combines to create a powerful incentive structure for authors to find certain results, and powerful incentives lead to biased decision making. For example, pharmaceutical companies have received much criticism for prioritising the publication of trials showing drugs to be highly effective, while delaying or suppressing the publication of data suggesting more modest effects [3, 17]. While financial incentives are an obvious source of bias in pharma, academics operating in such a competitive career culture may be equally at risk of bias. Indeed, the evidence produced in competitive research environments may be particularly unreliable, with the proportion of studies reporting positive results increasing with increased competition in US research institutions [12].

This pressure to publish in a publication system that favours positive results undermines scientific integrity, both by dissuading authors from publishing null findings, but also by incentivising researchers to adopt questionable research practices to maximise their chances of finding something positive, and thus more publishable, in each data set [12]. Flexible analytical procedures [38], especially in low-powered studies, can generate a large number of positive results, although most will either be false positive or inflated [5]. Researchers may incorrectly write these analyses up as if they were confirmatory tests, retro-fitting a new hypothesis to explain a chance result [22].

There are numerous ‘questionable research practices’ which authors can use to exploit the multiple decision points during data collection and analysis to generate positive results [22]. These include the removal of an outlier, transforming a variable, collecting more data, switching outcome variables, adding or removing covariates, until one happens upon a significant result [38]. Researchers may then forget about the unsuccessful paths, and write-up only those which yielded statistically significant results [29]. There is good evidence that such undisclosed flexibility in analysis is common practice. In a survey of 2000 psychologists, over half admitted to having failed to report all dependent measures, and selectively reporting studies that “worked”, with the estimated actual prevalence of these behaviours (using admission estimates) rising to nearly 100% [22].

Undisclosed analytical flexibility is a particularly insidious form of bias, as it resonates so deeply with the natural human desire for seeking and embellishing information that supports one’s beliefs and ignoring or discrediting that which does not [1, 26]. This, combined with the unintuitive nature of statistical inference, means that many a selective reporting error may be made in ignorance [4, 30]. However, the easier path to publication for manuscripts reporting strong, positive, consistent results, creates a strong incentive for researchers to find and selectively report such results. Therefore, while editorial decisions during peer review remain influenced by the nature of a study’s results, publication bias will persist as researcher behaviour will adapt accordingly.

### Initiatives to reduce publication bias and increase transparency

To reliably inform treatment decisions, social policies, or the design of the next incremental empirical study, the published literature must include all available data that is of acceptable quality [11]. While psychology and social sciences may have led the way in demonstrating and describing publication bias, medicine and, in particular, the systematic review and clinical trials movement, has since led the avocation and implementation of scientific practices to mitigate its effects. These include public repositories for the mandatory registration of trial protocols (e.g., ClinicalTrials.gov and ISRCTN), comprehensive guidelines for transparent reporting of procedures and results (the EQUATOR network), and the publication of study protocols.

#### Pre-registration

Registration of clinical trial protocols before data collection commences is now mandatory, making it possible to trace trials from inception to completion. In the UK, the National Institute for Health Research has gone a step further and made publication of results, in addition to protocol pre-registration, a legal obligation for all studies that they fund. However, although this ensures that the publication record is virtually complete, and that risk of bias in results from questionable research practices is reduced, the direction or strength of results may still bias reviewer and editorial decisions, such that, holding quality constant, null findings might end up in lower impact journals [27, 28], or may take longer to be submitted for publication at all [3].

Pre-registration of study protocols is a powerful tool against some forms of publications bias. The protocol repository provides an audit trail for studies, recording what should be present in a complete publication record and thus opening the file drawer. The inclusion of detailed analysis plans deters questionable research practices, highlighting where data exploration deviates from the planned test of the a-priori hypothesis. Following medicine’s example, several platforms supporting protocol publication have been launched to promote transparency in psychology and the social sciences (e.g., the Centre for Open Science’s Open Science Framework, and the Berkeley Initiative for Transparency in the Social Science (BITSS), to name just a few). Some journals, including the medical journals of the *BMC* series, also publish study protocol articles in an effort help to improve the standard of medical research, reduce publication bias and improve reproducibility.

### Solutions

A problem as thorny as the publication bias will require multiple interventions to resolve. However, a central aim must be to re-align incentives for career progression with those for conducting high-quality rigorous research. The peer-review process offers a relatively self-contained process during which this re-alignment might be achieved, by basing editorial decisions on the scientific rigour of study design alone. If publication is determined by judgements of study quality, then it is expected that researcher behaviour will adapt accordingly, but this needs to be measured empirically. There are multiple ways journals could shift reviewer and editorial decisions towards concerns of study quality.

#### Journals’ publishing ethos and guidance

Many journals provide guidance to encourage authors and reviewers to focus on assessing study quality. For example, in 2015, BioMed Central introduced a Minimum Standards of Reporting checklist for authors and reviewers [23]. The ethos of some open access journals, including the journals of the *BMC* series, explicitly state that editorial decisions are based solely on whether the work meets rigorous technical and ethical standards. However, if reviews are based on full papers which include results, any judgements about technical rigour are likely to be confounded by the results; as described above, methods from null studies will likely be judged as less rigorous, and the implications of methodological limitations as much greater. While such initiatives are a positive step to addressing publication bias, bias will inevitably persist, due to the powerful retrospective influence results have on how study quality is assessed.

Perhaps the only way to prevent the review and editorial process being influenced by a study’s results is to base the decision to publish solely on assessing the scientific merit of the study rationale, and the appropriateness and rigour of the proposed methods, without access to the results and discussion. This aligns the reward of publication with study quality. There are two routes by which this could happen. The first is where a commitment to publish is made before the study is begun, based on the strength of the study protocol, as in the case of Registered Reports [8]. The second is where a decision to publish is made after study completion in the usual manner, but where the results and discussion sections of the paper are withheld, and reviewers decide whether the study merits publication based solely on the background and methods sections.

#### Registered reports

The Registered Reports (RR) format was pioneered in the journal *Cortex* [8], and since been adopted by over 40 journals, from a regular publication option to issuing special issues (https://osf.io/8mpji/wiki). In RRs, the study protocol is submitted for peer review before any experiments are conducted and, if the protocol is deemed to have scientific merit, an editorial commitment is made, in advance, to publishing the outcomes. Armed with this provisional acceptance, authors can conduct the research safe in the knowledge that the results themselves will not determine the article's publication [9].

The RR format has many advantages; it allows for peer review at a point where reviewers can suggest key improvements to study design, rather than simply stating why the experiment is flawed. It also clearly prevents the results from biasing the decision to publish. However, RRs impose time restrictions and may delay the start of studies by several months while the protocol undergoes peer review. The practicalities of busy academic life mean that RRs are unlikely to fully replace the traditional review process. Academics likely have a range of scientific endeavours, and RRs may suit the timescale or more confirmatory nature of some studies, but not necessarily others in their portfolio. For example, the timing of final year undergraduate student projects may be difficult to fit into a RR format [6].

#### ‘Results-free’ peer review

As there will likely be a place for the traditional ‘post-study’ peer-review process for some time, a logical and relatively simple way to encourage editorial decisions to be based on a study’s methods is to blind reviewers and editors to the study’s results. This month, *BMC Psychology* launches a pilot to trial a new ‘results-free’ peer-review process, to address the bias in the editorial process. Editors and reviewers will be blinded to the study’s results, and decide whether to accept or reject manuscripts based on the scientific merits of their rationale and methods alone. Authors submit otherwise complete manuscripts, but omit any discussion of results, and provisional acceptance is based on peer review of the background and methods alone. The results and discussion of accepted manuscripts may then be reviewed in a second stage, to check for adherence to methods and to allow minor revisions.

This simple approach offers an eloquent solution to many of the key drivers of publication bias discussed above, and a recent pilot in a politics journal of a similar process [13] indicates that ‘results-free’ reviews are feasible, and acceptable to authors and reviewers, though the numbers were relatively small. ‘Results-free’ review should tackle bias that occurs during the actual review process, by preventing reviewer judgments of study quality being biased against studies with null results. It also incentivises authors to write up high-quality studies with null results, and might dissuade them from submitting low-quality studies with dubious positive results. Knowing that the reviewers will be focussing on the rationale and methods might also improve the quality and transparency of methods reporting. Thus, ‘results-free’ review has the potential to increase the transparency of methods reporting, improve the scientific quality of published research, and increase in the overall reliability of results.

### Evaluating the effectiveness of proposed solutions

There has been a proliferation in new publishing initiatives designed to reduce publication bias, and while this is laudable, it is important that these initiatives are systematically and rigorously evaluated to ensure they are having the desired outcomes. *BMC Psychology* is taking the bold step to conduct a randomised controlled trial to evaluate their ‘results-free’ peer-review process. In the first instance, a single arm pilot will assess the feasibility of ‘results-free’ review and optimise the process. Following this we plan to conduct a full randomized controlled trial to assess the effects of results-free review on publication bias and the editorial decision-making process, and collating author, editor, and reviewer feedback. If deemed feasible and effective, it is our hope that we may roll out results-free review (with any revisions) across other BioMed Central journals. We have designed the process to be as simple as possible, as an alternative model that can be integrated as part of the traditional review process, or more radically, to replace traditional post-study review if the evidence shows it to be superior. We welcome comments and feedback on the process as the trial progresses.

### Concluding remarks

Addressing a problem as thorny as the wider reproducibility crisis will require multiple interventions to resolve, but a central philosophy must be the re-alignment of incentives for career progression with those for conducting high quality rigorous research. Scientist should be encouraged to conduct and publish science of the highest scientific rigour and integrity, and this will only be achieved if editorial decisions are based on the methodological quality of the research rather than its outcomes. The results-free review model, launched this month in *BMC Psychology,* offers a solution by focusing editorial decisions on the scientific rigour of the study design, and preventing editorial decisions being unduly biased by study findings. The human powers of self-persuasion and post-hoc justification mean that withholding results from peer-reviewers may be the only reliable way to protect reviewers and editors against the often unconscious influence of the results justifying the means.

## References

[1] Beck AT (1976). Cognitive therapy and the emotional disorders.

[2] Begley CG, Ellis LM (2012). Drug development: Raise standards for preclinical cancer research. Nature.

[3] Bourgeois FT, Murthy S, Mandl KD (2010). Outcome reporting among drug trials registered in ClinicalTrials.gov. Ann Intern Med.

[4] Button KS. Statistical Rigor and the Perils of Chance. eNeuro. 2016;3(4). doi:10.1523/ENEURO.0030-16.2016. 10.1523/ENEURO.0030-16.2016 PMC4945734 27482537

[5] Button KS, Ioannidis JP, Mokrysz C (2013). Power failure: why small sample size undermines the reliability of neuroscience. Nat Rev Neurosci.

[6] Button KS, Lawrence NS, Chambers CD (2016). Instilling scientific rigour at the grassroots. Psychol.

[7] Button KS and Munafò MR. Powering Reproducible Research. In: Lilienfeld SO and Waldman ID (eds) Psychological science under scrutiny: Recent challenges and proposed solutions. New York: Wiley & Sons. (in press).

[8] Chambers CD (2013). Registered reports: a new publishing initiative at Cortex. Cortex.

[9] Chambers CD, Dienes Z, McIntosh RD (2015). Registered reports: realigning incentives in scientific publishing. Cortex.

[10] Coursol A, Wagner EE (1986). Effect of positive findings on submission and acceptance: A note of meta-analysis bias. Prof Psychol Res Parctice.

[11] Dickersin K (1990). The existence of publication bias and risk factors for its occurrence. JAMA.

[12] Fanelli D (2010). Do pressures to publish increase scientists' bias? An empirical support from US States Data. PLoS ONE.

[13] Findley MG, Jensen NM, Malesky EJ, Pepinsky TB. Can results-free review reduce publication bias? The results and implications of a pilot study. Comparative Political Studies. 2016;49(13):1667–1703.

[14] Fisher R (1955). Statistical methods and scientific induction. J Royal Stat Soc Series B-Stat Methodol.

[15] Francis G (2012). Publication bias and the failure of replication in experimental psychology. Psychon Bull Rev.

[16] Franco A, Malhotra N, Simonovits G (2014). Social science. Publication bias in the social sciences: unlocking the file drawer. Science.

[17] Goldacre B (2012). Bad pharma : how drug companies mislead doctors and harm patients.

[18] Greenwald AG (1975). Consequences of prejudice against the null hyptohesis. Psychol Bull.

[19] Ioannidis JP (1998). Effect of the statistical significance of results on the time to completion and publication of randomized efficacy trials. JAMA.

[20] Ioannidis JP (2005). Why most published research findings are false. PLoS Med.

[21] Ioannidis JPA (2012). Why Science Is Not Necessarily Self-Correcting. Perspect Psychol Sci.

[22] John LK, Loewenstein G, Prelec D (2012). Measuring the Prevalence of Questionable Research Practices With Incentives for Truth Telling. Psychol Sci.

[23] Kenall A, Edmunds S, Goodman L, et al. Better reporting for better research: a checklist for reproducibility. BMC Neurosci. 2015;16:44. 10.1186/s12868-015-0177-z PMC4512017 26202681

[24] Laws KR (2016). Psychology, replication & beyond. BMC Psychol.

[25] Leimu R, Koricheva J (2005). What determines the citation frequency of ecological papers?. Trends Ecol Evol.

[26] Mahoney MJ (1977). Publication prejudices: An experimental study of confirmatory bias in the peer review system. Cognit Ther Res.

[27] Munafo MR, Stothart G, Flint J (2009). Bias in genetic association studies and impact factor. Mol Psychiatry.

[28] Murtaugh PA (2002). Journal qulaity, effect size, and publication bias in meta-analysis. Ecology.

[29] Neuroskeptic (2012). The Nine Circles of Scientific Hell. Perspect Psychol Sci.

[30] Nuzzo R (2015). Fooling ourselves. Nature.

[31] Perel P, Roberts I, Sena E (2007). Comparison of treatment effects between animal experiments and clinical trials: systematic review. BMJ.

[32] Prinz F, Schlange T, Asadullah K (2011). Believe it or not: how much can we rely on published data on potential drug targets?. Nat Rev Drug Discov.

[33] Rosenthal R (1979). The File Drawer Problem and Tolerance for Null Results. Psychol Bull.

[34] Rothstein H, Sutton AJ, Borenstein M (2005). Publication bias in meta-analysis: prevention, assessment and adjustments.

[35] Schillebeeckx M, Maricque B, Lewis C (2013). The missing piece to changing the university culture. Nat Biotechnol.

[36] Scott S, Kranz JE, Cole J (2008). Design, power, and interpretation of studies in the standard murine model of ALS. Amyotroph Lateral Scler.

[37] Shadish WR, Doherty M, Montgomery LM (1989). How many studies are in the file drawer? an estimate from the family/marital psychotherapy literature. Clin Psychol Rev.

[38] Simmons JP, Nelson LD, Simonsohn U (2011). False-positive psychology: undisclosed flexibility in data collection and analysis allows presenting anything as significant. Psychol Sci.

[39] Smart RG (1964). The importance of negative results in psychological research. Canadian Psychol.

[40] Smith ML (1980). Sex bias in counseling and psychotherapy. Psychol Bull.

[41] Sterling TD (1959). Publication decisions and their possible effects on inferences drawn from tests of significance--or vice versa. J Am Stat Assoc.

[42] Steward O, Popovich PG, Dietrich WD (2012). Replication and reproducibility in spinal cord injury research. Exp Neurol.

[43] van Dijk D, Manor O, Carey LB (2014). Publication metrics and success on the academic job market. Curr Biol.

[44] Vankov I, Bowers J, Munafo MR (2014). On the persistence of low power in psychological science. Q J Exp Psychol (Hove).

[45] Yong E (2012). Replication studies: Bad copy. Nature.

